# Nelson-Salassa Syndrome Progressing to Pituitary Carcinoma: A Case Report and Review of the Literature

**DOI:** 10.7759/cureus.5595

**Published:** 2019-09-08

**Authors:** Lucas P Carlstrom, Christopher S Graffeo, Avital Perry, Janalee K Stokken, Jamie J Van Gompel

**Affiliations:** 1 Neurological Surgery, Mayo Clinic, Rochester, USA; 2 Otorhinolaryngology, Mayo Clinic, Rochester, USA

**Keywords:** nelson-salassa syndrome, nelson syndrome, cushing disease, pituitary carcinoma, bilateral adrenalectomy

## Abstract

Nelson-Salassa Syndrome (NSS) is a rare sequela of bilateral adrenalectomy as a treatment for persistent hypercortisolism in refractory Cushing disease (CD). Radiographic NSS has been observed in half of CD patients after adrenalectomy, yet often follows a mild course and rarely requires treatment. We present the case of a 44-year-old male with a history of CD who underwent primary treatments including transsphenoidal resection, radiotherapy, and bilateral adrenalectomy. He subsequently presented with acute vision loss and progressive somnolence. MRI revealed marked enlargement of an invasive sellar and suprasellar lesion exerting significant mass effect on the chiasm, and multiple new enhancing bony lesions. The patient was taken for emergent transsphenoidal resection and calvarial biopsy; visual function was restored postoperatively, and pathologic analysis confirmed pituitary carcinoma. While NSS typically follows an indolent course, pituitary carcinoma is a highly morbid metastatic disease, and has been theorized to occur at a higher frequency in the NSS population. We review all published cases of NSS to pituitary carcinoma progression, which further underscores the highly aggressive nature and considerable mortality of this patient cohort. Although mild, asymptomatic NSS is more commonly observed, symptomatic patients or those with rapid growth after adrenalectomy, should be targeted for routine close clinical follow-up and serial radiographic surveillance.

## Introduction

Nelson-Salassa Syndrome (NSS) is an uncommon neuroendocrine phenomenon following bilateral adrenalectomy as a treatment for persistent, symptomatic hypercortisolism in the setting of severe, refractory Cushing disease (CD). In NSS, adrenalectomy removes cortisol-mediated negative feedback on the adenoma remnant, which may rapidly progress and yield a large adrenocorticotropic hormone (ACTH)-secreting macroadenoma and, in the classic description, precipitating visual field deficits from chiasmatic compression, and skin hyperpigmentation from the marked increase in circulating alpha-melanocyte-stimulating hormone-a metabolic byproduct of ACTH synthesis. Interestingly, although radiographic evidence of NSS has been reported in up to 50% of post-adrenalectomy CD patients, the pathognomonic triad is rarely observed in practice [1-5]. Risk factors for NSS include young age, larger pituitary adenoma at time of adrenalectomy, and a history of external beam radiotherapy (EBRT). Although potentially challenging to control medically, most cases of NSS undergo an indolent course that never requires further intervention; however, rare cases of anaplasia have been described [1, 6-15].

By contrast, pituitary carcinoma (PC) is an exceedingly rare, invasive manifestation of the most aggressive pituitary adenomas; although the cancerous diagnosis arises in 0.2% of adenomas, it carries a one-year mortality of 66% [9, 16]. The defining feature of PC is extra-sellar spread via hematogenous or other modalities of dissemination that are distinct from local invasion. PC is typically recognized as aggressive early in the disease process, and characterized by poor responses treatment with a propensity for rapidly, robust recurrence that portends subsequent metastasis [17]. ACTH-secreting adenomas comprise the largest subgroup of PC, accounting for approximately 40% of all reported cases [16]. The underlying pathophysiologic mechanism driving transformation is unknown, as is the nature of the relationship between ACTH-secreting tumors and PC. Less often, indolent adenomas have been observed to metastasize years after primary treatment; among these cases, presentation is delayed a mean 15 years from adenoma diagnosis to PC [9]. However, in spite of the associations between phenotypically aggressive adenoma behavior and NSS, the present case represents only the third report of PC arising in association with NSS-a rapidly fatal combination.

## Case presentation

**Figure 1:**
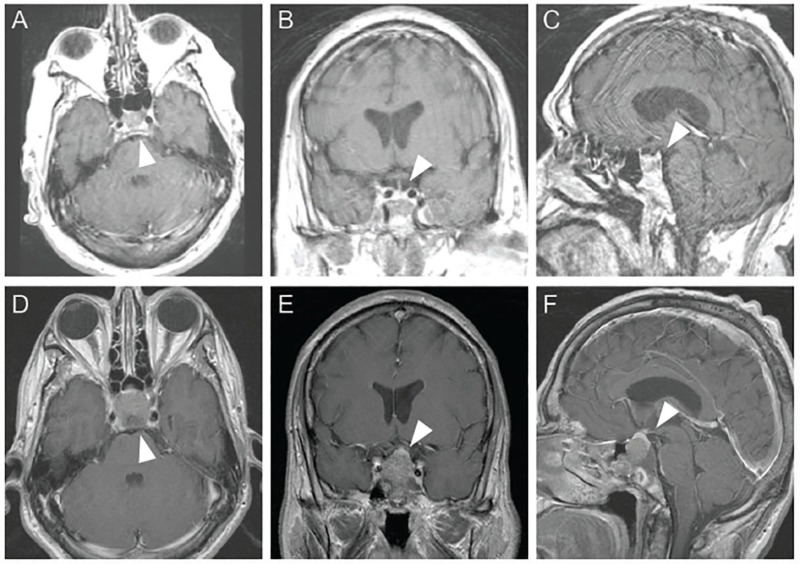
The patient presenting with Cushing disease Gadolinium-contrasted T1 brain MR images in axial (A), coronal (B) and sagittal (C) planes demonstrating no clear evidence of a microadenoma and showing possible fibrous dysplasia or other benign fibro-osseous lesion. Due to clinical deterioration, the patient underwent repeat MRI one year later revealing a new sellar lesion - axial (D), coronal (E), and sagittal (F). White arrows designate sellar and parasellar region of interest.

A 44-year-old gentleman underwent initial endocrinologic evaluation at an outside facility five years prior to presentation at our institution for recurrent episodes of bacteremia, systemic infections, and cutaneous hyperpigmentation. Laboratory investigations revealed 24-hour urine cortisol of 1968 ug/24-hr, morning serum cortisol of 55 ug/ml, evening serum cortisol of 59 ug/ml, and serum ACTH of 169 pg/ml. High-dose 8 mg dexamethasone suppression testing resulted in a morning serum cortisol of 46 ug/ml and serum ACTH 268 pg/ml, inconsistent with pituitary ACTH hypersecretion. Abdominal and pelvic computed tomography (CT) scans were unremarkable for an ectopic source, and so subsequent disease progression prompted brain magnetic resonance imaging (MRI), which was negative for microadenoma interpreted as possible fibrous dysplasia or other benign fibro-osseous lesion (Figure 1A-1C). As the patient was severely symptomatic despite optimal medical management and no evidence of a clear source for the abnormal hypercortisolism, bilateral adrenalectomy was recommended. Adrenal pathology demonstrated hyperplastic glands without neoplastic cells. Over the following year, worsening symptoms lead to a repeat brain MRI that revealed a new sellar lesion (Figure 1D-1F), which was subsequently treated with two transsphenoidal operations and EBRT at 50.4 Gy in 28 fractions. In review of these outside images, there is abnormal signal within the clivus that may represent local sellar lesion spread.

**Figure 2:**
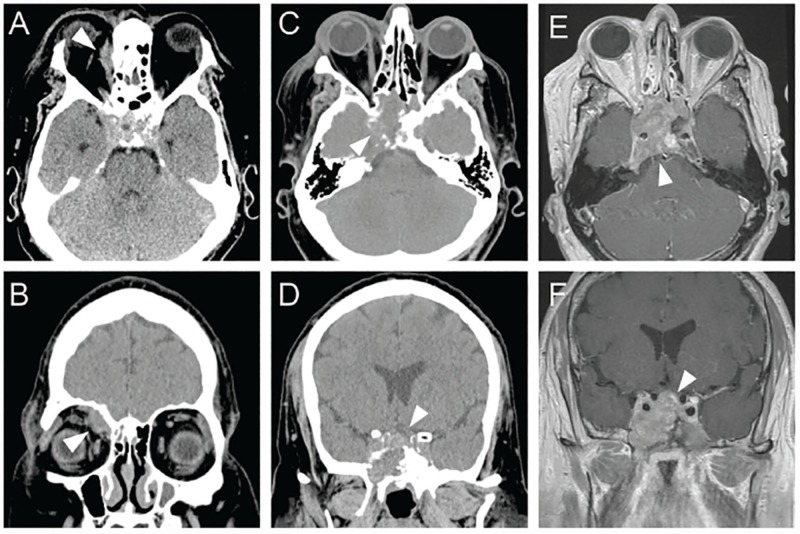
Patient progression from Cushing disease Non-contrasted axial (A) and coronal (B) tissue-windowed head CT images highlighting right superomedial right orbital abscess (arrowheads). Non-contrasted axial (C) and coronal (D) tissue windowed head CT images and gadolinium-contrasted T1-weighted axial (E) and coronal (F) brain MR imaging revealing invasive pituitary macroadenoma with suprasellar extension and local bony destruction (arrowheads).

**Figure 3:**
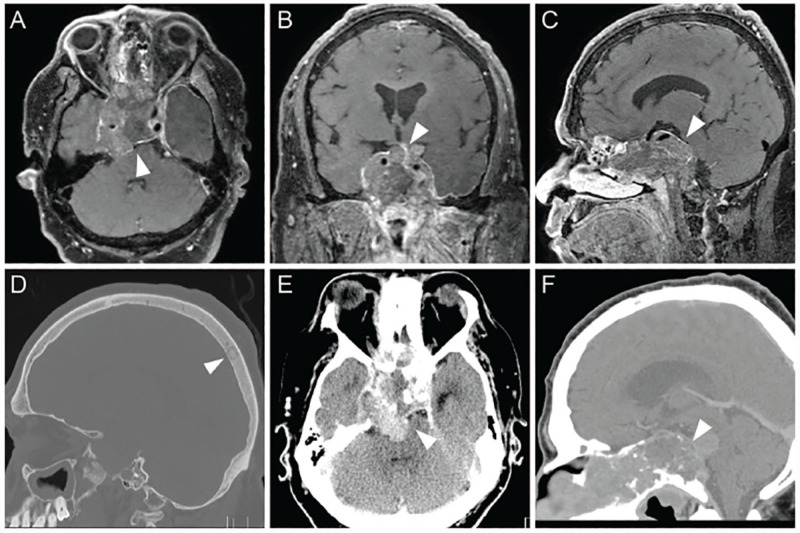
Patient transition to pituitary carcinoma Gadolinium-contrasted T1 brain MR images in axial (A), coronal (B) and sagittal (C) planes demonstrating enlarging invasive macroadenoma (arrowheads). Sagittal non-contrasted head CT in bone window reveals sclerotic calvarial lesion (arrowhead) (D). Non-contrasted head CT imaging revealing continued invasive growth of the pituitary adenoma around the clivus and prepontine cistern, precipitating marked brainstem compression, axial (E) and sagittal (F) (arrowheads).

Two years following the appearance of a pituitary lesion, the patient developed new right retro-orbital pressure and periorbital swelling, with CT imaging revealing a lesion in the superomedial right orbit with periosteal reaction concerning for subperiosteal abscess (Figure 2A-2B). Bilateral endoscopic maxillary antrostomies and bilateral total ethmoidectomies with right anterior orbitotomy were carried out for surgical debridement, with subsequent histopathologic studies demonstrating chronic inflammation and numerous eosinophils. Follow-up head CT and brain MRI one month later at an outside facility identified an invasive sellar lesion with suprasellar extension at which time he was referred to our institution. Prior to his appointment, the patient presented to our emergency department with acute right-sided headache and ptosis (Figure 2C-2F). Imaging demonstrated progression of the sellar mass with new nasal cavity involvement; however, the patient declined admission, and was lost to follow-up for several months, until he again presented to our emergency department, now with new diplopia attributable to right cranial nerve III and VI palsies and an incomplete left intranuclear ophthalmoplegia. Repeat MRI confirmed persistent right orbital abscess with comorbid enlarging invasive macroadenoma (Figure 3A-3C) and newly identified calvarial lesions (Figure 3D). Empiric broad spectrum antibiotics coverage was initiated, but the following morning, the patient developed acute right monocular vision loss, associated with an ophthalmic artery filling defect on computed tomography angiography (CTA) that prompted urgent transsphenoidal debulking and stereotactic biopsy of a calvarial lesion.

**Figure 4:**
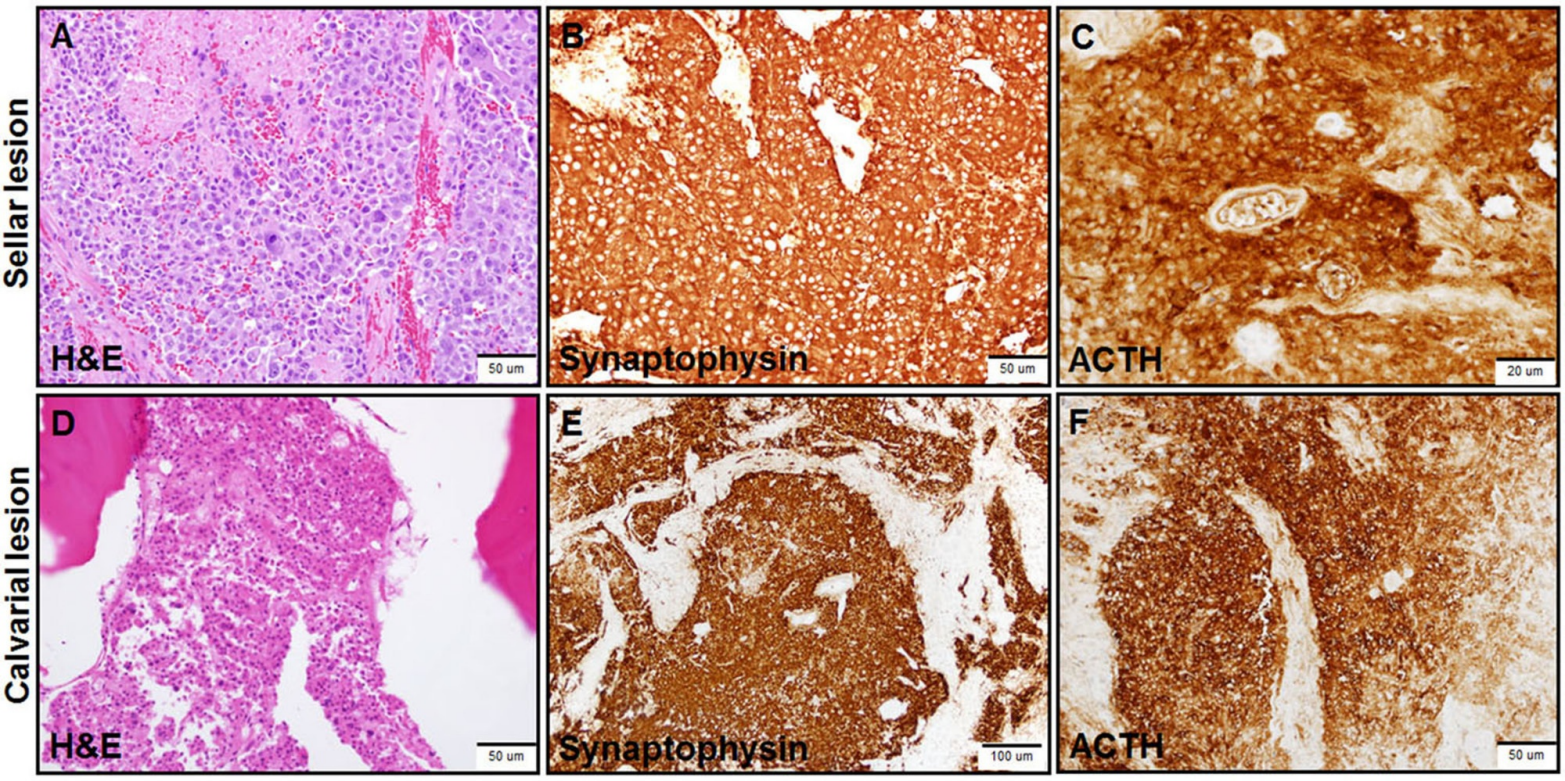
Histologic analysis of the pituitary carcinoma Immunohistochemical assessment using hematoxylin and eosin, synaptophysin, and ACTH stains of the sellar lesion tissue (A-C) and the calvarial lesions (D-F) at the time emergent surgical debulking and exploration following acute monocular vision loss confirmed ACTH-producing pituitary carcinoma lesions. H&E stain: Hematoxylin and eosin stain; ACTH: Adrenocorticotropic hormone.

Post-operatively, vision was restored, while histopathology of the calvarial lesion identified an ACTH-immunoreactive neuroendocrine tumor with pituitary immunophenotype, confirming PC (Figure 4A-4F). The patient was subsequently discharged with a recommended palliative radiation, but he declined further interventions. Six months later, the patient returned to the emergency department with blurred vision, headache, and shaking chills. Head CT revealed evidence of destructive, expansive, and invasive growth of the lesion involving the bilateral cavernous sinuses, paranasal sinuses, clivus, and prepontine cistern, precipitating marked brainstem compression and acute obstructive hydrocephalus (Figure 3E-3F). Chest, abdomen and pelvic CT scans further discovered likely metastatic disease in bilateral femurs, spine, peri-renal space, and ribs. The patient elected to pursue palliative measures only, and died of disease four months later.

## Discussion

NSS is a complex and enigmatic neuroendocrine condition, the diagnosis and optimal management of which has been variably described in the literature, with the most recent and rigorous recommendations proposing radiographic criteria that consequently highlighted both the high prevalence and potential indolence of the disease sequela [1]. The risk factors predisposing to the development of NSS after adrenalectomy are poorly understood, but generally thought to include phenotypically aggressive CD. Although aggressive CD is frequently conceptualized as those patients with the largest, fastest growing, or most severely symptomatic adenomas, an interesting and oft overlooked CD cohort that may be at particularly high risk of NSS are those individuals who presented with symptomatically severe disease but initially negative neuroimaging studies, as in the present case. Few studies have specifically examined these patients; however, the propensity for a small population of neoplastic cells to generate extreme quantities of ACTH and therefore downstream corticosteroids may be an important index and an area for further investigation in the future-particularly when considering tumors that biochemically behave like true carcinomas even before the diagnosis of PC, as demonstrated by the lack of the expected response on high-dose dexamethasone suppression testing [18, 19]. With this in mind, we advise consideration for frequent MRI screening in all post-adrenalectomy patients with NSS, and in particular those who had negative pre-adrenalectomy sellar MRI studies - however, the appropriate long-term surveillance intervals remain an area for further study.

NSS progression to PC was first reported by Dr. Robert Salassa in 1959, and has been reported in a few sporadic reports over the following decades [5, 9-15]. Among patients presented with PC at a median of 42 years of age (range 27-69 years), 66% were women (n = 10). Maximum recorded serum ACTH levels varied widely (80-280,000 pg/ml), as did NSS management, with treatments including combination surgical resection and radiotherapy (n = 5, 33%), radiotherapy alone (n = 5, 33%), repeat surgery alone (n = 2, 13%), radiotherapy followed by stereotactic radiosurgery (n = 1, 7%), and two patients in which no further treatment was undergone. Progression from NSS to PC confirmed a median five years (range 1-31) after diagnosis of NSS; metastatic sites included the spinal subarachnoid space, posterior fossa, and liver, other intracranial sites, bone, and lung which were treated with medical palliation, surgery, radiotherapy, or a combination of surgery and radiation. Overall median follow-up was one year after diagnosis of PC (range 7 days-4 years), with an overall survival rate of 27% (n = 4) at the time of last follow-up. Patients that underwent surgical treatment for NSS were deceased at a median of six months following PC diagnosis - likely indicating more aggressive lesions. Patients that underwent radiation treatment for NSS had a median time at last follow-up of 1.25 years - with three patients being alive at last record. There was no statistical difference in time from NSS diagnosis to progression to PC between patients that underwent radiation treatment (median 5, range 1-31) and those that did not (median 7.5, range 4-12) (p = 0.73), however, the case volume is so low it is difficult to make meaningful conclusions. It remains unclear how radiation treatment may impact progression to PC or local tumor control, and in particular if in our patient contributed towards tumor control. A larger cohort would have to be amassed to make any substantive claims about the impact of radiation, or any treatment modality, on disease progression in NSS-PC cases (Table 1).

**Table 1:** NSS to pituitary carcinoma literature review Literature review of all documented cases of Nelson-Salassa Syndrome progressing to pituitary carcinoma. NSS: Nelson-Salassa Syndrome; PC: Pituitary carcinoma; na: not available; RT: Radiation treatment; SA: Subarachnoid space; PF: Posterior fossa; DC: Deceased; d: day; m: month; yr: year; cyclophos: cyclophosphamide; 5-FU: fluorouracil.

Author	Year	Age (years)	Sex	ACTH levels (pg/ml)	NSS treatment	Carcinoma type	NSS to PC diagnosis (years)	Metastatic site	Treatment of metastases	Follow-up
Salassa et al. [5]	1959	42	M	na	surgery, RT	na	4	Extracranial	None	5 m - DC
Kaiser et al. [10]	1983	27	F	230,000	RT	ACTH	5	Liver, lung, mediastinum, bone	Cyclophos, adriamycin, 5-FU	3 yr
Papotti et al. [11]	1984	32	F	1020	RT	ACTH	1	SA, 4th ventricle, brain stem	Brain stem, meninges	2 yr - DC
Gabrilove et al. [12]	1986	37	M	>2500	RT	ACTH	3	Spinal cord, cauda equina, heart, liver, pancreas	None	2 m - DC
Pernicone et al. [9]	1997	37	M	na	RT, surgery	ACTH	17	Spinal SA, liver	Surgery, RT	7 d - DC
Pernicone et al. [9]	1997	48	F	280,000	RT	ACTH	18	Bone (multiple)	RT	1.5 yr
Pernicone et al. [9]	1997	55	F	na	Surgery	ACTH	4	Spinal SA, liver	None	5 m - DC
Pernicone et al. [9]	1997	69	M	1800	Surgery, RT	ACTH	11	Cerebellum, parietal lobe, spinal SA	RT	1 yr - DC
Kemink et al. [13]	1999	25	F	110,000	Surgery, RT	ACTH	31	Temporoparietal region	None	1 yr - DC
Scheithauer et al. [14]	2001	35	F	9605	na	ACTH	12	Liver	Surgery	3.5 yr
Scheithauer et al. [14]	2001	59	F	33,000	na	ACTH	7	PF, spinal SA	Surgery	4 yr - DC
Gaffey et al. [15]	2002	59	F	200	RT, SRS	ACTH	5	Liver	Surgery, cyclophos, vincristine, dacarbazine	2 yr
Gaffey et al. [15]	2002	35	F	80	RT	ACTH	4	Posterior fossa, spinal	Surgical decompression, RT	4 yr - DC
Gaffey et al. [15]	2002	63	F	-	Surgery	ACTH	8	Temporoparietal region	Surgery, RT	-
Carlstrom et al. [1]	2019	44	M	268	Surgery, RT	ACTH	5	Calvarium	None	10 m - DC

In contrast to the frequently indolent neoplasia of NSS, PC is a universally aggressive and highly morbid malignancy that carries a mean survival of two years from diagnosis to death-a prognosis that is further reduced in the presence of metastasis outside the central nervous system (CNS) [17]. Overall, the most common locations for PC metastasis include the spinal subarachnoid space and posterior fossa within the CNS, and the liver outside of the CNS. Although the invasion of the intracranial venous system and large calvarial metastatic burden in our patient indicated a high potential for systemic dissemination, body imaging was never pursued, in large part due to his decision to elect palliation once metastatic evidence of PC was discovered.

Although based on a small sample, preceding reports described a median time-to-progression of 11 years from NSS to PC, more than double of our patient’s progression within five years. The degree to which this is attributable to poor treatment compliance is difficult to estimate; notwithstanding, the dramatic tumor expansion witnessed particularly in the late stages of his disease highlights the capacity to PC to take on a violent clinical trajectory. Indeed, once diagnosed, PC is often a harbinger of impending mortality, particularly in the setting of NSS, after which the median survival time drops to 10 months - less than half the grim 24 months of survival anticipated in PC that develops without preceding NSS.

Detailed pathologic information has rarely been reported in NSS-PC, with Scheithauer et al. providing a long description of a patient’s metastatic liver disease, in which the malignant cells appeared larger, with more typical secretory granules, and with increased nuclear polymorphism, as compared to the primary sellar lesion [14]. Our patient’s metastatic calvarial lesions were positive for synaptophysin, chromogranin, cytokeratin AE1/3, and ACTH, without Crook cells-effectively an identical morphology to the primary sellar lesion. However, the impact non-CNS metastatic disease has on mortality in PC patient remains unclear due to the rarity of the pathology and heterogeneity of the disease manifestations. Characterizing pathology and collection of clinical data from additional metastatic PC lesions should help shed additional light on this facet of PC disease.

Given the paucity of preceding reports, establishing screening criteria or predictive factors for prognostication or stratified monitoring has not been possible, apart from the weak association between macroadenomas that simultaneously invade the cavernous sinus and suprasellar space - a phenotypic growth pattern that, while often noted among those patients with PC, occurs in exponentially more patients who never undergo malignant transformation [20]. One theory put forward to explain this association is that cases involving both these anatomic spaces are highly likely to have both residual disease and some degree of iatrogenically empowered communication between the sella and either the subarachnoid or intravenous spaces, promoting tumor dissemination [9]. Interestingly, Pernicone et al. reported the longest latency interval to PC, 18 years, which was noted in a patient who never underwent cranial surgery; consequently, one can speculate that, even though this patient’s tumor was transformed at some point during its history, the absence of iatrogenic destruction of the diaphragm sella or other encapsulating structures may have delayed the eventual metastasis. Although inadequate detail was provided in that report to draw a more complete picture, the comparison to our own case suggests that local invasion may be the precursor to metastatic spread in patients without a surgical tract providing ready access to the subarachnoid or intravenous compartments.

## Conclusions

NSS is a complex disease process that, although more prevalent than previously estimated, is typically described by an indolent clinical course. Notwithstanding, rare cases may progress to PC, with both intra- and extracranial metastases, in which circumstances the preceding NSS diagnosis portends a dramatically more aggressive clinical course, often resulting in mortality within a year. The invasive and highly malignant nature of PC renders surgical management effectively palliative; however, there is a defined role for decompressive interventions in many patients, whose quality of life may be significantly restored by neurosurgical treatment of PC, as in our patients, whose vision was saved for the four months between PC diagnosis and death. Given the dramatic capacity for grievous outcomes in PC, we recommend close follow-up and routine MRI surveillance of patients with NSS for evidence of on-going progression or metastasis, as early intervention likely provides the only potential for improved outcomes in this exceedingly rare and deadly disease.
